# Hydroclimatological variability and dengue transmission in Dhaka, Bangladesh: a time-series study

**DOI:** 10.1186/1471-2334-12-98

**Published:** 2012-04-24

**Authors:** Masahiro Hashizume, Ashraf M Dewan, Toshihiko Sunahara, M Ziaur Rahman, Taro Yamamoto

**Affiliations:** 1 Institute of Tropical Medicine (NEKKEN) and the Global Center of Excellence program, Nagasaki University, 1-12-4 Sakamoto, Nagasaki, 852-8523, Japan; 2 Department of Geography and Environment, University of Dhaka, Ramna 1000, Dhaka, Bangladesh; 3 Department of Spatial Sciences, Curtin University of Technology, Kent Street, Bentley 6102, Perth, Australia; 4 Department of Sociology, University of Dhaka, Ramna 1000, Dhaka, Bangladesh

**Keywords:** Bangladesh, Climate, Dengue, River level, Time-series

## Abstract

**Background:**

While floods can potentially increase the transmission of dengue, only few studies have reported the association of dengue epidemics with flooding. We estimated the effects of river levels and rainfall on the hospital admissions for dengue fever at 11 major hospitals in Dhaka, Bangladesh.

**Methods:**

We examined time-series of the number of hospital admissions of dengue fever in relation to river levels from 2005 to 2009 using generalized linear Poisson regression models adjusting for seasonal, between-year variation, public holidays and temperature.

**Results:**

There was strong evidence for an increase in dengue fever at high river levels. Hospitalisations increased by 6.9% (95% CI: 3.2, 10.7) for each 0.1 metre increase above a threshold (3.9 metres) for the average river level over lags of 0–5 weeks. Conversely, the number of hospitalisations increased by 29.6% (95% CI: 19.8, 40.2) for a 0.1 metre decrease below the same threshold of the average river level over lags of 0–19 weeks.

**Conclusions:**

Our findings provide evidence that factors associated with both high and low river levels increase the hospitalisations of dengue fever cases in Dhaka.

## Background

Dengue fever is a mosquito-borne infection that causes potentially fatal complications like dengue haemorrhagic fever (DHF) and dengue shock syndrome. The global incidence of dengue has increased dramatically in recent decades. About 2.5 billion people living in tropical and subtropical urban and semi-urban areas are now at risk, and over 50 million cases of dengue are estimated to occur annually [1]. In some Asian countries, DHF is a leading cause of hospitalisation and death in children [2].

In Bangladesh, sporadic cases of dengue fever were documented between 1964 and 1999. The first outbreak of DHF occurred in Dhaka in 2000 [3] and since then, cases have been reported every year with clear seasonality, suggesting that weather factors could play a role, either directly or indirectly.

Climatic conditions directly affect the biology of the vector mosquitoes, *Aedes aegypti* and *Aedes albopictus*[4-6]. High rainfall and temperatures can provide the conditions for oviposition, stimulation of egg-hatching, high vector development and a decrease in the reproductive period of the virus in the mosquito [4-6]. Many studies have investigated the relationship between climate and dengue in various locations. High temperatures have been associated with dengue in Brazil [8], China [9], Costa Rica [10], Indonesia [11], Mexico [12], Puerto Rico [13], Singapore [14], Taiwan [15] and Thailand [16] and high rainfall has been associated with dengue in Barbados [17], Indonesia [18], Mexico [12], Puerto Rico [13], Taiwan [15], Thailand [19], Trinidad [20] and Venezuela [21]. It has been suggested that spatial heterogeneity of the short-term associations between cases of dengue and temperature and rainfall may be attributed to underlying climate heterogeneity [13]. Some studies have suggested an association between dengue epidemics and El Niño [22-24].

Floods can potentially increase the transmission of dengue. Standing waters caused by the overflow of rivers can act as breeding sites for mosquitoes, and thereby enhance the potential for exposure of flood-affected populations to dengue [25]. However, few studies have reported an association of dengue epidemics with flooding. No dengue infections were reported in relief workers in Puerto Rico after hurricane Georges (1998) [26,27] and no outbreaks of dengue occurred after hurricane Jeanne (2004) in Haiti [28]. Only a low incidence of dengue was reported in flood-affected areas of Malaysia [29]. After flooding in Thailand, an increase in the number of acute pyrexia cases, of which 29% were dengue and 27% were leptospirosis, was reported [30].

Bangladesh is a low-lying country with bodies of water that are vulnerable to flooding. Clarification of the potential role of river levels and weather on the transmission of dengue could help provide insights into the pathways of seasonal epidemics of the disease and improve disease control. This study aimed to estimate the effect of river level and rainfall on the incidence of dengue in Dhaka while controlling for other seasonal determinants.

## Methods

### Hospital data

**Figure 1:**
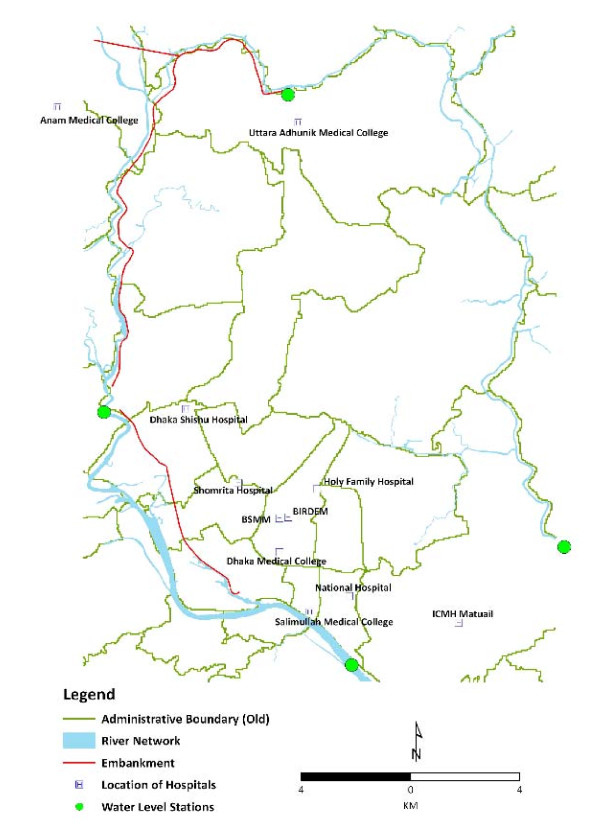
Locations of the 11 hospitals and river level monitoring stations in Dhaka, Bangladesh that were used in this study.

The primary outcome for this study is the weekly number of patients admitted to 11 principal hospitals in the Dhaka Metropolitan area and diagnosed with dengue fever and DHF. Figure 1 shows the locations of the 11 hospitals. These hospitals were selected because they are the major health service providers in the Dhaka Metropolitan area. Data for patients admitted between January 2005 and December 2009 were obtained from their hospitalisation records. A database was developed to document patient data, including the date of admission. Diagnosis of dengue fever was made by physicians in the hospitals and some, but not all, diagnoses were confirmed by laboratory investigation.

### River level and meteorological data

The daily river levels at 4 monitoring stations (Buriganga River at Mill Barrack, Tongi Khal at Tongi, Turag River at Mirpur and Balu River at Demra) in Dhaka (Figure 1) were obtained from the Bangladesh Water Development Board. The daily average of the maximum river levels at the four stations was used in the analysis. We also obtained the daily rainfall and mean temperature data in Dhaka from the Bangladesh Meteorological Department. Weekly means of the daily maximum river levels at the four stations and of the mean daily temperature, as well as the total weekly rainfall were calculated from the daily records.

### Statistical analysis

We examined the relationship between the number of weekly dengue cases and river levels and weather variables (temperature and rainfall) using generalized linear Poisson regression models allowing for overdispersion [31]. To account for the seasonality of dengue counts not directly related to the river levels and weather, Fourier terms up to the 5th harmonic were introduced into the model. Fourier terms can capture repeated periodic (e.g., seasonal) patterns comprising a combination of pairs of sine and cosine terms (harmonics) of varying wavelengths [32]. The number of harmonics was chosen as Akaike’s Information Criteria (AIC). Indicator variables for the years of the study were incorporated into the model to allow for long-term trends and other variations between years. An indicator variable for public holidays was included in the model to control bias in the event that holidays affected access to hospital. To allow for the autocorrelations an autoregressive term of order 1 was also incorporated into the model [33].

To account for delays in the effect of the river levels on the number of dengue cases, lagged river level variables were built-in to the model. We considered lags (delays in effect) of up to 26 weeks (6 months). To identify the optimum lag period, a linear term for river levels for each lag (0, 1, 2, …, 26 weeks) was sequentially incorporated into a model comprising indicator variables of years and public holidays and Fourier terms (i.e., a model with no controls for weather variables). To create a distributed lag, lags were added 1 week at a time up to the lag of interest [34]. The approximately linear increase in the effect in each additional lag was observed up to a lag of five weeks (i.e., a six-week period between a given week and the preceding five weeks), while a linear decrease in the effect was observed between lags of 6 –19 weeks. The optimal lag for a high river level effect was chosen when the effect was at its maximum, from zero to five weeks (the average of the river levels on a given week and the five previous weeks), and the optimal lag for a low river level effect was chosen when the effect was at its minimum, from 0 to 19 weeks.

In the initial analyses, designed to identify the broad shape of any association, we fitted natural cubic splines (3 df) [35] to (a) the average river level over lags 0–19 weeks, and (b) the average river level over lags 0–5 and 6–19 weeks, as separate splines simultaneously included in the model. We have incorporated all variables of rainfall, temperature and river level as a natural cubic spline (3 df) with the same lag period in the final model to adjust for potential mutual confounding. A detailed description of the final model is given in the supplementary material.

Because the initial analyses suggested a log-linear association, we fitted a linear threshold model, comprising linear terms for river level. Guided by the spline analysis, we based the low and high river level terms on the 0–19 and 0–5 week averages, respectively. The choice of thresholds was based on the maximum likelihood estimation for the river level over a grid of all possible one-decimal point values within the range indicated on the river level-dengue graphs, and constrained for interpretability so that low threshold (η_l_) = high threshold (η_h_) where unconstrained estimates gave η_l_ > η_h_. Likelihood profile confidence intervals (CIs) for the threshold were calculated as the thresholds for which deviance of the model was 3.84 more than the minimum. An increase or decrease in the number of cases that were associated with a 0.1 metre increase or decrease in a given measure of river levels, estimated as coefficients from the regression model, was reported as percentage change.

Using the simple threshold model, we examined the lag effects in more detail by fitting linear unconstrained distributed lag models comprising terms for low and high river level at each lag in the preceding 19 weeks [34].

To investigate whether the main results were sensitive to the levels of control for seasonal patterns, the analyses were repeated using Fourier terms up to the 12th harmonic per year adding one harmonic at a time (0–10 pairs of harmonics). Instead of Fourier terms, indicator variables for each month were also examined. To investigate the sensitivity of the main results, the river level data from each of the monitoring stations were analysed separately.

All statistical analyses were carried out using Stata version 10.0 (Stata Corporation, College Station, Texas).

### Ethics Statement

This study has been approved by the ethics committee of the University of Dhaka and all data analyzed were anonymized.

## Results

**Figure 2:**
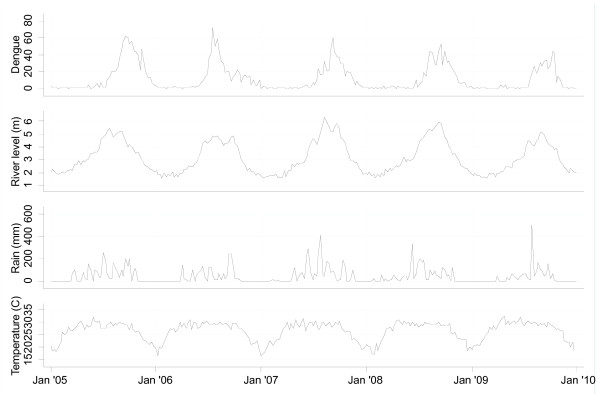
Seasonal variations in the number of dengue cases per week, and river level, temperature and rainfall data in Dhaka, 2005–2009.

**Figure 3:**
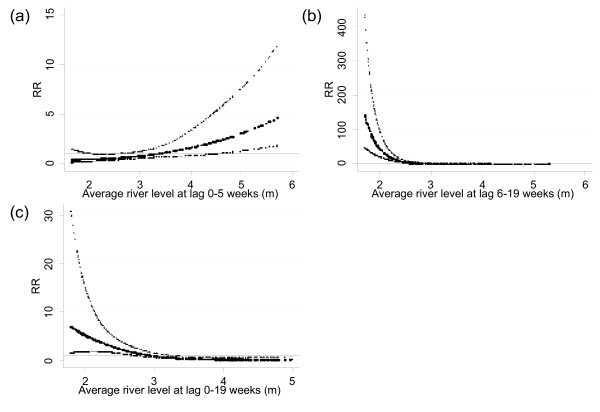
**Relationship between the number of dengue cases and average river levels over lags of (A) 0–5 weeks, (B) 6–19 weeks and (C) 0–19 weeks (shown as a 3 df natural cubic spline) adjusted for seasonal variation, interannual variations, public holidays, temperature and rainfall.** RR represents the relative risk of dengue (scaled against the mean weekly number of cases). The centre line in each graph shows the estimated spline curve, and the upper and lower lines represent the 95% confidence limits.

There were 3130 admissions for dengue fever to the 11 hospitals from 2005 to 2009. Descriptive statistics for the number of patients and weather variables are displayed in Table 1. Dengue fever had a single peak at the end of the monsoon (weeks 29–40) following a lag of 0–13 weeks with the peak river level (Figure 2). The relationships between the number of dengue fever cases and river level, adjusted for season, interannual variations, holidays, temperature and rainfall are shown in Figure 3. An increase in dengue fever was seen with high river levels at lag 0–5 weeks, and an increase in the number of cases with low river levels was observed at lag 6–19 weeks. An increased number of dengue cases with low river levels were observed at lag 0–19 weeks. Maximum likelihood estimates of the threshold for high and low river levels coincide at 3.9 metres (95% CI: 3.7–4.0) for average river levels over lags of 0–5 and 0–19 weeks calculated using a double thresholds model. For a 0.1 metre increase above the threshold, the number of dengue fever cases increased by 6.9% (95% CI: 3.2–10.7). For a 0.1 metre decrease below the 3.9 metre average river level at lag 0–19 weeks, the number of cases increased by 29.6% (95% CI: 19.8–40.2).

**Figure 4:**
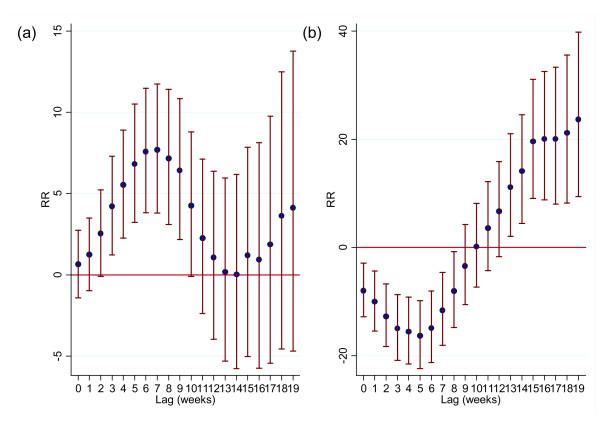
**Percent change (and 95% CIs) in the number of dengue cases for (A) “high” river level (per 0.1 m increase above threshold) and (B) “low” river level (per 0.1 m decrease below threshold) at each lag (unconstrained distributed lag models).** The results shown are from preliminary models adjusted for seasonal variation, interannual variations and public holidays.

In the distributed lag model, a “high river level” effect was observed at shorter lags with the highest estimate at a lag of six weeks followed by lower estimates in later lags (Figure 4A). In contrast, the positive effect of a low river level was observed at longer lags; the estimate was lowest at a lag of five weeks followed by an increased effect up to a lag of 19 weeks (Figure 4B).

**Table 1:** Distribution of daily number of dengue patients admitted to 11 hospitals and the average of the meteorological and river level data in Dhaka, 2005-2009

**Variable (unit)**	**Mean**	**SD**	**Minimum**	**Maximum**
Dengue fever	12.0	15.9	0	72
River level (m)				
Maximum	3.16	1.27	1.51	6.33
Minimum	2.90	1.39	1.13	6.28
Rainfall (mm)	45.1	71.5	0	497
Temperature (°C)				
Mean	26.6	3.8	16.5	32.4
Maximum	31.0	3.3	21.0	37.6
Minimum	22.3	4.6	11.1	28.8

**Figure 5:**
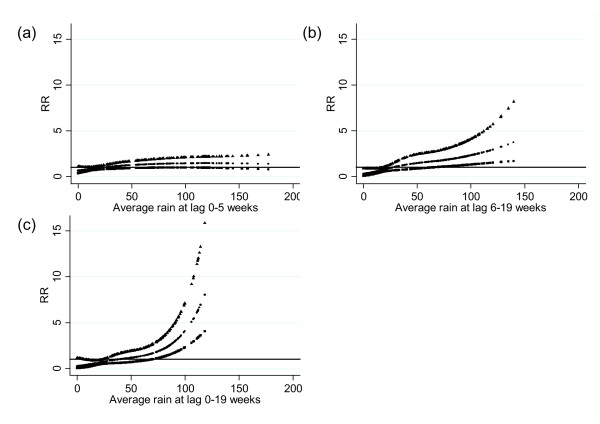
**Relationship between the number of dengue cases and average rainfall over lags of (A) 0–5 weeks, (B) 6–19 weeks and (C) 0–19 weeks (shown as a 3 df natural cubic spline) adjusted for seasonal variation, interannual variations, public holidays, temperature and river level.** RR represents the relative risk of dengue (scaled against the mean weekly number of cases). The centre line in each graph shows the estimated spline curve, and the upper and lower lines represent the 95% confidence limits.

The relationship between the number of dengue fever cases and the rainfall adjusted for season, interannual variations, holidays, river level and temperature is shown in Figure 5. There was no obvious association at lag 0–5 weeks, while the pattern showed a positive slope with high rainfall at lag 0–19 weeks, and a possible threshold at around 60–80 millimetres of rain. The estimated threshold was 70 millimetres (95% CI: 59–79). For a 10 mm increase above the threshold, the number of cases increased by 29.0% (95% CI: 14.0–46.0). The relationship between the number of dengue fever cases and temperature was also examined but no statistically significant association was observed. AIC and model deviances for combinations of independent variables are displayed in Additional file 1: Table S1. The minimum AIC value was for model 3, which considered rain and river level as covariates.

When the analyses were repeated using river level data from each of the 4 monitoring stations separately, similar patterns for the effects of high and low river level were obtained. For the four individual stations, the high river level slope varied from 2.8% to 7.9% above the same threshold (3.9 metres) and the low river level slope varied from 6.7% to 25.3% below the threshold.

In the sensitivity analyses, when the degree of seasonal control was varied from two to ten harmonics, the estimates of the effect of high and low river levels changed little while the estimates decreased when no seasonal control was built-in to the models ( Additional file 1: Figure S 1).

## Discussion

In this study, we found a significant association between hospital visits for dengue and high river levels with a short lag time (0–5 weeks) and with low river levels with a longer lag time (6–19 weeks) in Dhaka, Bangladesh. The results indicate that the number of dengue cases increased when a prolonged low river level preceded a high river level. This result is consistent with a report that found that the number of DHF cases was higher when prolonged drought preceded the rainy season [36].

Previous reports had indicated that the incidence of dengue rarely increases after water-related disasters such as floods and hurricanes [26-29]. Flood water may create stagnant pools or fill containers already in the environment. Stagnant ground pools except for sewage water and septic tanks [37,38], however, are not a common larval site for the container-inhabiting *Aedes* mosquito and flood water caused by overflows of river water may not be of optimal quality for the development of *Aedes* larvae. Flooding in Dhaka is not caused by flash floods. Levels in water bodies scattered throughout the city gradually increase every monsoon season, and river levels can be good indicators of the level of water bodies in the community irrespective of the overflow of the major rivers. Thousands of pieces of garbage, including plastic containers, are scattered along water bodies. When water remains in discarded containers after the increased water levels recede, breeding conditions for the *Aedes* mosquitoes that are capable of spreading dengue may be created. Eggs of *Aedes* mosquitoes are desiccation-resistant and are commonly laid above the waterline in tree holes, tires or other water-holding cavities [39]. When dry conditions prevail during the previous 6–19 weeks, water levels in the cavities gradually decrease and the eggs end up at varying distances above the waterline as a result of several ovipositions at different times. Thus, eggs laid by different female mosquitoes will accumulate in a cavity as the water level drops (and the area of the inner wall above the water increases); this is one of the cumulative effects of low water levels. The eggs will hatch when submerged in water as a result of an increase in the level of water bodies [40]. Our hypothesis is supported by a previous report that a large number of *Aedes albopictus* was identified in a flooded area [41]. Mechanistic models to estimate *Aedes* mosquito abundance in response to flooding have shown that forcing by flooding is able to underpin changes in Aedes mosquito population dynamics [42,43]. *Aedes albopictus* is more likely than *Aedes aegypti* to breed and transmit dengue outside the home [40], and it is the principal vector of dengue transmission in Dhaka [44]. Dhaka has unique topographical characteristics where the low-lying land and abundant bodies of water may be related to the observed association between river level and dengue incidence. Further environmental and entomological studies are necessary to elucidate the causal pathways of these associations.

Intensive and integrated control by source reduction, chemical control (larviciding and adulticiding) and health promotion were reported to minimize the adverse effects of flooding [29]. Individual preventive behaviours like sleeping in air-conditioned rooms and wearing long-sleeved clothing were also reported to reduce the chance of mosquito bites [27]. Thus, appropriate public health interventions can minimize the number of dengue cases even after water-related disasters. Insufficient control measures like source diminution, insecticide spraying and health promotion potentially contribute to the flood-associated increase in dengue cases in Dhaka. In addition, recent urbanisation and population growth, especially in the slum areas of Dhaka, may have increased the city’s susceptibility to flood-associated dengue.

There are some limitations to this study. First, we used the aggregated number of dengue cases from 11 hospitals as the database, and the average river level of four rivers surrounding Dhaka city as the indicator of water levels in the community. There could be discrepancies between the residential areas and flood-affected areas. Because this is a random misclassification model, the estimates found in this study could be underestimated. The estimates of the effect may be more precise if the exposure of the cases was measured based on the river level at the monitoring station closest to the domiciles of the cases. Second, we used hospitalisation data from the principal hospitals in Dhaka. The hospitals included in the study may not cover all residents in the city; however, they are the main referral hospitals in the city and, therefore, the most severe cases are likely to have been included. Third, the diagnoses of dengue fever were made by physicians and not all of the diagnoses were confirmed in the laboratory. The differential diagnosis of dengue from leptospirosis is especially important because the clinical symptoms of dengue and leptospirosis are similar [45]. Although, after the first outbreak of dengue fever in Dhaka in 2000, physicians in the hospitals included in this study were generally well trained in differential diagnosis [46], there is still a possibility of ascertainment bias in the study. Fourth, we did not examine the effect of population immunity on the models. There are 4 serotypes (DEN-1, DEN-2, DEN-3 and DEN-4) of the genus *Flavivirus* and, while individuals acquire permanent immunity to each serotype infecting them, there is no evidence of cross-immunity [39]. However, we consider that population immunity will not materially alter the results because immunity to re-infection does not change over time and it is unlikely to have obscured the short-term (less than 6 months) associations between dengue and the factors investigated in this study.

## Conclusions

No vaccine is yet available for dengue and there is no specific treatment; so that dengue control is primarily dependent on and a function of the control of the *Aedes* mosquito. Thus, the development of an early warning system is an important public health goal. The integration of climate or hydrological data into predictive frameworks for dengue has not yet been realized. Our study provides the basis for the early prediction of dengue epidemics and has the potential to improve disease control.

Because systematic mosquito data for the study area were not available, the findings of this study do not represent a causal connection. However, this study points to the possibly important role of river levels on predicting dengue incidence in Dhaka. Further studies that incorporate entomological information are warranted.

## Competing interests

The authors have no competing interests.

## Authors’ contributions

AMD and MH designed the study. AMD collected data. MH analyzed data. MH wrote the paper. All authors have been involved in revising the manuscript critically for important intellectual content, and have given final approval of the version to be published. All authors read and approved the final manuscript.

## Pre-publication history

The pre-publication history for this paper can be accessed here:

http://www.biomedcentral.com/1471-2334/12/98/prepub

## Supplementary Material

Additional file 1 **Table S1.** Diagnostics of dengue-rainfall/river level models. **Figure S1.** Sensitivity analysis. Percent change (and 95% CIs) in the number of dengue cases for “high” (A; per 0.1 m increase above threshold) and “low” river level (B; per 0.1 m decrease below threshold) with each number of harmonics and indicator variable of month (M). Presented results are from final models adjusted for seasonal variation (5 harmonics), interannual variations, public holidays, temperature and rainfall. Click here for file
