# Supplementary material for: Hydroclimatological variability and dengue transmission in Dhaka, Bangladesh: a time-series study
Source: BMC Infect Dis. 2012 Apr 24;12:98. doi: 10.1186/1471-2334-12-98 (PMC3528427; doi:10.1186/1471-2334-12-98)
Supplement: Additional file 1 — Table S1. Diagnostics of dengue-rainfall/river level models. Figure S1. Sensitivity analysis. Percent change (and 95% CIs) in the number of dengue cases for “high” (A; per 0.1 m increase above threshold) and “low” river level (B; per 0.1 m decrease below threshold) with each number of harmonics and indicator variable of month (M). Presented results are from final models adjusted for seasonal variation (5 harmonics), interannual variations, public holidays, temperature and rainfall. [file 1471-2334-12-98-S1.doc]

**SUPPLEMENTARY ON-LINE MATERIALS**

**Models for river level**

Terminology:

The variables “river level”, “temp” and “rain” indicate average weekly river level and temperature and mean weekly amount of rainfall in each lag, respectively. NS indicates a natural cubic spline function. Fourier represents Fourier (trigonometric) terms. i.year represents indicator variables of year. i.holiday represents an indicator variable for weeks that include public holidays. (x)^+^ = x if x>0, otherwise = 0.

Model 1: spline for river level over lags 0−19 weeks (figure 3-c):

*log[E(Y)] = NS(river level_0−19_, 3 df) + (confounders)*

Model 2: splines for river level over lags 0−5 and 6−19 weeks (figures 3-a and 3-b):

*log[E(Y)] = NS(river level_0−5_, 3 df) + NS(river level_6−19_, 3 df) + (confounders)*

Model 3: linear-threshold model (Text slope estimates):

*log[E(Y)] = β_low_(low_threshold-river level_0−19_)^+^ + β_high_(river level_0−5_,-high_threshold)^+^ + (confounders)*

Model 4: distributed lag model for high and low river level (figure 4)

*log[E(Y)] = Σ β_low,l_(low_threshold-river level_i-l_)^+^ + β_high_(river level_i-l_-high_threshold)^+^ + (confounders)*

Confounder terms (in all models for river level):

*(confounders)= α+ time(Fourier, 5 harmonics/year) + i.year + i.holiday +*

*(for Model 1) NS(temp_0−19_, 3 df) + NS(rain_0−19_, 3 df)*

*(for Models 2 and 3) NS(temp_0−5_, 3 df) + NS(temp_6−19_, 3 df) + NS(rain_0−5_, 3 df) +*

*NS(rain_6−19_, 3 df)*

*(for Model 4) nothing*

Reference List

DerSimonian R, Laird N. Meta-analysis in clinical trials. Control Clin Trials 1986;7:177-88.

| Table s1. Parameter estimates of the final model (Model 3 in the supplementary online materials) | | | | | | |
| --- | --- | --- | --- | --- | --- | --- |
|  |  |  |  |  | 95% Confidence interval | |
| Variable | Coefficient | s.e. | z | p value | Lower limit | Upper limit |
| low river level^*^ | 2.5926680 | 0.4013654 | 6.46 | 0.000 | 1.8060060 | 3.3793290 |
| high river level^**^ | 0.6628930 | 0.1784555 | 3.71 | 0.000 | 0.3131267 | 1.0126590 |
| temp0-5 | 0.0277317 | 0.3833508 | 0.07 | 0.942 | -0.7236222 | 0.7790855 |
| temp0-5sp1 | -0.0033825 | 0.0155889 | -0.22 | 0.828 | -0.0339362 | 0.0271711 |
| temp0-5sp2 | 0.0074288 | 0.0377827 | 0.20 | 0.844 | -0.0666239 | 0.0814816 |
| temp6-19 | -0.2658805 | 0.4674096 | -0.57 | 0.569 | -1.1819870 | 0.6502256 |
| temp6-19sp1 | 0.0049987 | 0.0149294 | 0.33 | 0.738 | -0.0242624 | 0.0342597 |
| temp6-19sp2 | -0.0307667 | 0.0391163 | -0.79 | 0.432 | -0.1074332 | 0.0458999 |
| rain0-5 | 0.0442173 | 0.0161235 | 2.74 | 0.006 | 0.0126160 | 0.0758187 |
| rain0-5sp1 | 0.0000263 | 0.0000128 | 2.05 | 0.040 | 0.0000012 | 0.0000515 |
| rain0-5sp2 | -0.0000038 | 0.0000022 | -1.70 | 0.088 | -0.0000082 | 0.0000006 |
| rain6-19 | 0.0493941 | 0.0291335 | 1.70 | 0.090 | -0.0077064 | 0.1064946 |
| rain6-19sp1 | 0.0000095 | 0.0000151 | 0.63 | 0.528 | -0.0000200 | 0.0000390 |
| rain6-19sp2 | -0.0000010 | 0.0000041 | -0.24 | 0.809 | -0.0000090 | 0.0000070 |
| year_2006 | -0.0427090 | 0.1414383 | -0.30 | 0.763 | -0.3199230 | 0.2345051 |
| year_2007 | -1.0707940 | 0.1350214 | -7.93 | 0.000 | -1.3354310 | -0.8061573 |
| year_2008 | -0.7312177 | 0.1154737 | -6.33 | 0.000 | -0.9575420 | -0.5048935 |
| year_2009 | -1.1236470 | 0.2232239 | -5.03 | 0.000 | -1.5611580 | -0.6861359 |
| holiday | -0.1072991 | 0.0767188 | -1.40 | 0.162 | -0.2576653 | 0.0430670 |
| cosine1 | 1.1036340 | 0.7775467 | 1.42 | 0.156 | -0.4203291 | 2.6275980 |
| sine1 | -1.6091420 | 1.0133890 | -1.59 | 0.112 | -3.5953480 | 0.3770634 |
| cosine2 | 0.2742382 | 0.4471101 | 0.61 | 0.540 | -0.6020815 | 1.1505580 |
| sine2 | 0.2494354 | 0.3509488 | 0.71 | 0.477 | -0.4384116 | 0.9372824 |
| cosine3 | 0.0675602 | 0.1064534 | 0.63 | 0.526 | -0.1410847 | 0.2762052 |
| sine3 | -0.2697735 | 0.2598513 | -1.04 | 0.299 | -0.7790726 | 0.2395256 |
| cosine4 | 0.0380518 | 0.1294794 | 0.29 | 0.769 | -0.2157232 | 0.2918268 |
| sine4 | -0.0748566 | 0.1687694 | -0.44 | 0.657 | -0.4056385 | 0.2559253 |
| cosine5 | -0.0090744 | 0.0761908 | -0.12 | 0.905 | -0.1584055 | 0.1402568 |
| sine5 | -0.0551522 | 0.0811509 | -0.68 | 0.497 | -0.2142050 | 0.1039006 |
| autoregressive lag 1 | 0.0958329 | 0.0171964 | 5.57 | 0.000 | 0.0621285 | 0.1295372 |
| constant | 1.4458470 | 13.8960000 | 0.10 | 0.917 | -25.7898100 | 28.6815000 |

*linear term below the threshold of 3.9 meter for the river level at lag 0–19 weeks

**linear term above the threshold of 3.9 meter for the river level at lag 0–5 weeks

Table s2. Diagnostics of dengue-rainfall/river level models

| Variable in the model | AIC | Deviance | Res. Df* | (1/df) Deviance |
| --- | --- | --- | --- | --- |
| river level | 5.26 | 481.0 | 230 | 2.091 |
| rain | 5.63 | 577.6 | 230 | 2.511 |
| rain+river level | 5.21 | 457.1 | 224 | 2.041 |

^*^Residual degrees of freedom

Models include a linear term and natural cubic splines of mean temperature and river level and/or rainfall, Fourier terms of 5 harmonics and indicator variables of years.

(A) (B)

Figure s1.

Sensitivity analysis. Percent change (and 95 % CIs) in the number of dengue cases for “high” (A; per 0.1 m increase above threshold) and “low” river level (B; per 0.1 m decrease below threshold) with each number of harmonics and indicator variable of month (M). Presented results are from final models adjusted for seasonal variation (5 harmonics), interannual variations, public holidays, temperature and rainfall.
